# Dissociative experiences and their relationship to mood problems among Arab mothers of disabled children

**DOI:** 10.11604/pamj.2013.15.21.2229

**Published:** 2013-05-12

**Authors:** Muwafak Al-Eithan, Hathab Al Juban, Asirvatham Alwin Robert

**Affiliations:** 1 Department of Psychology, Prince Sultan Military Medical City, Riyadh, Saudi Arabia; 2 Al-Imam Muhammad Ibn Saud Islamic University, Riyadh, Saudi Arabia; 3 Research Center, Sultan Bin Abdulaziz Humanitarian City, Riyadh, Saudi Arabia

**Keywords:** Dissociation, Mood, Depression, anxiety, mothers of disabled children, Saudi Arabia

## Abstract

**Introduction:**

To examine the presence of dissociation among Arab mothers with disabled children in Saudi Arabia, and to explore if this is linked to their mood difficulties, in addition to certain other demographic variables.

**Methods:**

We conducted a prospective study during the period of June 2011 to February 2012, on 86 mothers (study group) caring for children with physical, mental or sensory disabilities treated at Sultan Bin Abdulaziz Humanitarian City, Riyadh, Saudi Arabia. Patients’ selection was conducted using convenience sampling, non-probability technique. A total of 32 mothers (control group) with healthy children were also included. The Dissociative Experiences Scale (DES-11 Arabic) was used to measure dissociation whereas; the hospital anxiety and depression scale (HADS) was used to measure the mood symptoms of mothers. The demographic data of mothers and children were also collected.

**Results:**

The mean age of the children with disability was 5.6.±3.1 and healthy children 6.3±3.7 (range 1-14) years. The mean age of mothers in the study group (n=86) was 33.9±6.1 while the control group was 35.2±7.3 years. Results showed that the study group had higher level (Mean=39.9; SD=24.033) of dissociative experience than control sample (Mean=21.08; SD=14.487) (p=0.0001). Compared to control, mothers of disabled children scored significantly higher HADS-anxiety (p=0.042) and HADS-depression (p=0.021). In addition, results also showed that dissociation had significant correlation with mother's depression and anxiety. However, no significant correlations were found between dissociation and child's and mother's age.

**Conclusion:**

Mothers of disabled children in Saudi Arabia showed significantly more dissociative experiences than controls, which is correlated to their anxiety and depression. Clinical implications are discussed.

## Introduction

Dissociation is a complex psychophysiological process that arises on a continuum ranging from minor normative dissociations such as daydreaming to psychological conditions, dissociative identity disorder (DID), a chronic and poly-symptomatic condition formerly called multiple personality disorder (MPD) [1]. The literature is very informative of adults and children who develop such psychological disorder after a stress such as post traumatic distress disorder (PTSD) [1, 2]. People with PTSD, for instance, experience very unpleasant feelings or distress of recurrent involuntary images of distressful nature, or have disturbed sleep or intrusion of thoughts [1, 2]. The essential feature of dissociative disorders is a distraction in the generally integrated functions of consciousness, memory, identity, or perception of the environment [2]. The disadvantage of dissociative experiences is both psychological, cognitive and neuronal above that- there has been an indication that it effects higher neuronal circuits and consequently effect, memory, left hemisphere, integration of bodily sensations, sense of self, and affect and motivation [3].

The birth of a disabled child could induce complex feelings in both or one of the parents. There is rejection, shock, aggression, sadness and even lack of acceptance [4]. Some parents also experience a “psychological shock”. Several times, mothers even decline screening for disabilities in the antenatal period as they would not desire this reality to dawn upon them [5]. Feelings of guilt, depression, anxiety are all part of the adjustment process although some of the mothers adjust well, psychopathology remains rampant among the others [6]. All the above could lead some parents to experience features of distress and dissociation.

Studies reported that child with a disability may cause stress for parents who have the responsibility of undertaking every day care of their children, often even after their disabled children become adults [7]. The stigma of the disability and the prolonged grief of the parents regarding child's limited development and parents’ worries about the future of their children with disability, all add to parents’ emotional burden [8]. Naturally, it is expected that such parents with long term stress due to caring for disabled child, would have more physical and psychological problems like dissociation compared with parents of healthy children [9, 10]. The nature of the disability itself is a factor contributing to the challenges faced by such parent of disabled child and the way they cope and deal with it [11]. Studies reported elevated rates of depressive symptoms and feelings of increased psychological distress have been reported by mothers of children with chronic illness or disabling conditions [12]. Very little research has been carried out on caregivers of mothers or parents with disabled children, and we are not aware-if any- of published Arabic data on dissociation and the burden of caring for a disabled child.

In our previous studies, we reported the presence and a degree of mood problems in mothers with disabled children [13] and alexithymia [14]. In this present study we aimed to examine if mothers with disabled children suffered from features of dissociation and how this might be related to other mood disorders.

## Methods

### Study setting and sample

We conducted a prospective study during the period of June 2011 to February 2012, on 86 mothers (study group) of children with physical, mental or sensory disabilities treated at Sultan Bin Abdulaziz Humanitarian City in Riyadh, Saudi Arabia. A control group consisted of a 32 mothers (control group) with healthy children. The study was approved by the Research and Ethics Committee of Sultan Bin Abdulaziz Humanitarian City, Riyadh, Saudi Arabia. Written informed consent was obtained from all mothers. Patient's participation is voluntary and the data collected during the study has been handled confidentially. Mothers of disabled children's (≥ 1 year and ≤ 14 years) are considered to have a physical, mental or sensory disability was included in this study. Mothers with severe or chronic medical conditions (such as stroke, and diabetes mellitus), history of psychological disorder, and acute medical condition within the last 12-months were excluded from this study. The control group was recruited from personal contact (community), were informed that we were conducting a study in the Sultan Bin Abdulaziz Humanitarian City about the impact of the illness in the families and that we needed families without physical or psychical pathology as a control group.

The child was classified to have sensory problem if she/he suffer from hearing or vision difficulties. Mental disabilities referred to cognitive/intellectual difficulties (including language problems). Physical disabilities cover all motor problems and impaired activities of daily living (ADL) caused by diseases such as Cerebral Palsy (CP). The treating consultant (physiatrist) diagnosed all children, and who gave specific diagnosis of children's type of disability.

### Measures

The Dissociative Experiences Scale-II [15] is widely used test to measure the degree of such abnormal psychological experience, by clinical and research populations. The test is simple paper and pencil task, where the subjects rate their frequencies (from 0 to 100%) of having experiencing certain abnormal psychological experiences. A total score of 30 or above to identify those who may have severely dissociative. The normal procedure of translation and back translation carried out and showed very good similarities to the original English version. A small study of reliability of the scale was carried out on 10 mothers. The scale has showed reasonable reliability degree on repeated measure (Chronbach =.67).

The mood symptom's measurements were assessed for the mothers using Hospital Anxiety and Depression Scale (HADS), Arabic version.16 This mood scale is very simple and easy to use by most people with no major language problems, and has no cultural or psychological sensitive questions. In addition, it is known to have very high validity and reliability. The HADS consists of 7 items for anxiety (HADS-A) and 7 for depression (HADS-D). The items were scored on a 4-point scale from zero (not present) to 3 (considerable). The item scores are added, giving sub-scale scores on the HADS-anxiety and the HADS-depression from 0-21 [16]. The demographic data of mothers and children were also collected.

### Statistical analyses

Statistical analysis data analyses were carried out using Microsoft Excel 2002 (Microsoft Corporation, Seattle, WA) and SPSS 16 Program Package (SPSS Inc, Chicago, IL). The Dissociation, Anxiety and Depression data presented as mean ± SD. Student's t test and Pearson's correlations performed for the analyses.

## Results

The mothers of 53 (61.6%) male and 33 (38.4%) female children participated in the study. The mean age of the children with disability (study group) was 5.6.±3.1 and healthy children (control group) 6.3±3.7 (range 1-14) years. There were no significant differences in age of disabled and healthy children. The mean age of mothers in the study group (n=86) was 33.9±6.1 and the control group (n=32) was 35.2±7.3 years. There were no significant differences between the study and control groups in terms of mother's age either.

In the study group, 66.3% (n=57) of children had physical disability followed by 11.6% (n=10) mental disability, 10.5% (n=9) physical with mental disability, 2.3% (n=2) physical with sensory disability, 1.1% (n=2) mental with sensory disability, 4.7% (n=4) had combined disability i.e. physical, mental and sensory disability and only 3.5 (n=3) had sensory disability. A total of 59.3% of children's duration of disability was 0-4 years followed by 20.9% (n=18) 5-7 years and 19.8% of children's duration of disability was ≥ 8 years. Around 47.7% (n=41) mothers were university educated and 52.3% (n=45) under university educated. There were 86% (n=74) of the mothers were having a disabled child and 14% (n=12) mothers having 2 or more than 2 disabled children.

**Figure 1:**
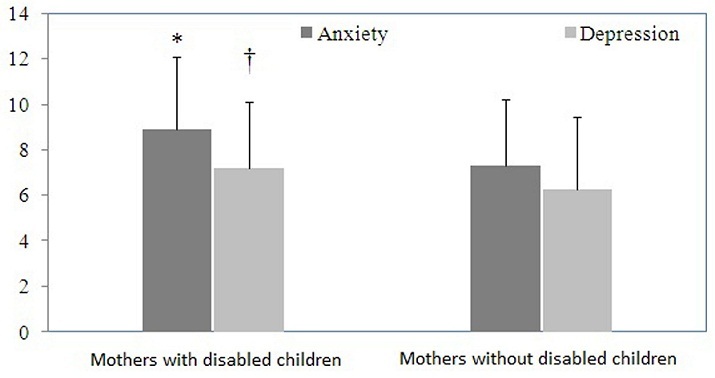
The differences of anxiety and depression among Arab mothers of disabled children

**Figure 2:**
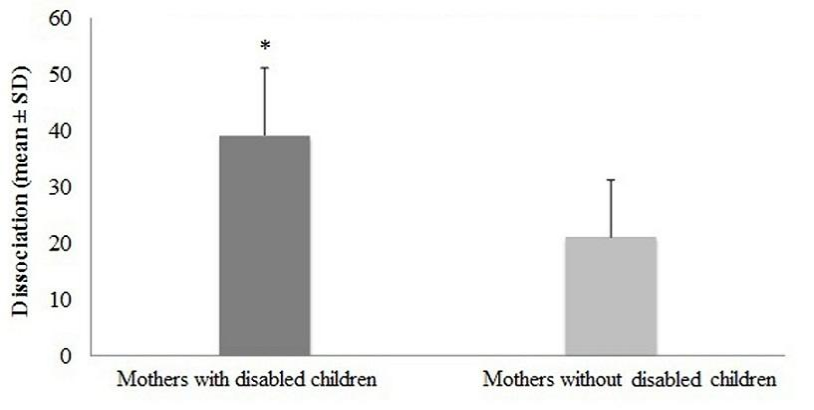
The differences of dissociative experiences (DES II) among Arab mothers of disabled children

The anxiety and depression results of mothers are shown in Figure 1. Compared to control group, mothers of disabled children had a significantly higher anxiety (p=0.042) and depression (p=0.021). The comparison of dissociation in control mother and study group is shown in Figure 2. The outcomes showed that mothers of disabled children had significantly higher degree of dissociation than control mothers (p=0.001). The correlation of dissociation, anxiety, depression, child age, mother age, duration of disability are shown in Table 1. The mothers’ dissociative experience is significantly correlated with anxiety, depression. However, dissociation was not correlated with child age, mother age and duration of disability (p> 0.05). Also there were no significant correlation were found in dissociation and anxiety, depression among control mothers (p> 0.05).

**Table 1:** Correlation of dissociation, depression, anxiety and age variables in the study group

Variables	dissociation	Anxiety	*Depression*	*Child age*	*Mother age*	*Duration*
Dissociation	1					
Anxiety P Value Df	.201 .019 84	1				
Depression P Value Df	.365 .000 84	.682 .000 84	1			
Child Age P Value Df	.105 .178 .84	−0.081 .232 84	−0.003 .490 84	1		
Mother Age P Value Df	−0.052 .304 84	−.243 .006 84	−.125 .101 84	.437 .000 84	1	
Duration P Value Df	.165 .151 84	−0.14 .904 84	.143 .200 84	.732 .000 84	.312 .004 84	1

Person's correlation: *p*-values <0.05 were taken as statistically significant (95% confidence interval)

## Discussion

With the birth of disabled child, parents were reported to experience a complex feeling that includes the feeling of losing someone beloved. It is an event that affects all the family members and their internal and external relationships [6]. The reaction to a loss has patterns of shock, denial, deal, depression, and acceptance-adjustment in adults [17]. Daily care routine, economic problems, receiving appropriate help and education are the basic hardships of the parents of a disabled child. Diagnostic confusions, behavioral and health problems, and feeling of loneliness in parents also add to these hardships [18, 19]. The increase in the severity of the disability results in a more dependent child, more responsibility for the parents, and more anxiety and distress in the parents [20].

Studies showed that the parents of an autistic child (for example) experienced over anxiety due to social relatedness, delay or absence of speech development, stereotypic movements, hyperactivity, and lack of eye contact [21, 22]. The mothers of autistic children were reported to be more introverted and neurotic than the normal control group [23] and the parents of children with autism and Down's syndrome were reported to be over-anxious, over-sensitive, stern in manner, and sensitive to be frustrated with criticism [24]. Studies of parents with disabled children suggested that 35-53% of mothers of children with disabilities passed cut-off scores for depression [25]. A previous study on Saudi mothers of disabled children have shown mood problems compared with controls [13]. In this present study we also found that mothers with disabled children possess more anxiety and depression as compared to control mothers.

The main question of this study was whether mothers of disabled children show features of dissociative experiences. The study found that when compared to control mother, mothers caring for disabled children showed significantly higher dissociation score on the DES-II (Arabic). Further, their dissociative experience is significantly correlated with anxiety, depression. However, dissociation was not correlated with child age and mother age. This is not surprising since burden of caring is indicated in other studies regardless of disability. The strong point of the study is that, it is the first time we examined the dissociative experiences in Arabic mothers caring for disabled children. The second question was related to the notion that dissociation might also show correlation to mood problems. This is also a positive result confirming our clinical observation. The current study may also add to this debate.

Theoretically, we are addressing the point of chronic (presumed-not acute) stress may enhance the chances of these mothers experiencing dissociation. Logically presented, the mothers with disabled children developed dissociation; and chronicity in the sense that mothers having to deal with disability of the child and much care needed accordingly (mood problems) after their experience with caring for disabled children- which it is in itself a stressful experience. The stress here is possible both traumatic and chronic. Traumatic stress is in the sense of having “bad news” about the child's disability (soon or long after giving birth), their caring for children with disability (as compared with mothers with healthy children) as chronic distress. Here, we assumed that at least chronic stress and burden of caring for a disabled child can cause some mothers to experience dissociative experience, but this may be sub-clinical presentation. Therefore, it is often neither investigated nor explored.

The study needs to improve on the scale validly and has better norms before final conclusion to be made. The study may have slightly smaller controls. In addition study may need to validate dissociative with other psychosomatic features as indicated in the early research development. Of course we have to consider the nature of the dissociative experience as a self-report and continuum concept, which may be limiting the categorization of cases vs. non-cases (those who experience dissociation and those do not). We assume that these mothers caring for disabled children have many stages in their long journey of the burden. They experience both acute and chronic distress as such. Clinically, mothers are not paid enough attention and often too late. The distress they experience may cause them to have such abnormal psychological features such as dissociation problems, with its psychological (e.g. mood problems) and physical problems (e.g. poor sleep). Needless to say here how important to attends to mothers′ well-being. Specially now there is a good and justified trend in rehabilitation and general health services aiming at looking after the carers themselves not only disabled persons or children.

## Conclusion

In conclusion, the findings of this study indicated that mothers of disabled children report dissociation experiences more than controls, in addition to anxiety and depression compared to their counterpart. Our findings suggest that more attention be needed to mothers’ psychological status, to enable them to support and improve rehabilitation strategies at home. Future research on similar population will consider their coping and the stress that they may go through due to the burden of caring for a child with disability. Clinical and administration recourses naturally and logically have to be placed for such mothers.

## References

[1] Tasman A, Goldfinger S.M Dissociative phenomena. Review of psychiatry.

[2] American Psychiatric Association (1994). Diagnostic and statistical manual of mental disorders.

[3] Zoroglu S, Yargic LM, Tutkun H (1996). Dissociative identity disorder in childhood: five Turkish cases. Am J Psychiatry..

[4] Quine L, Pahl J (1987). First diagnosis of severe handicap: a study of parental reactions. Dev Med Child Neurol..

[5] Gokhale LS, Cietak KA (2002). Serum screening for anomalies in pregnancy: reasons for acceptance or refusal of the test. J Obstet Gynaecol..

[6] Taanila A, Syrjala L, Kokkonen J (2002). Coping of parents with physically and/or intellectually disabled children. Child Care Health Dev..

[7] Bilgin S, Gozum S (2009). Reducing burnout in mothers with an intellectually disabled child: an education programme. J Adv Nurs..

[8] Seltzer MM, Greenberg JS, Floyd FJ (2001). Life course impacts of parenting a child with a disability. Am J Ment Retard..

[9] Seltzer MM, Greenberg JS, Floyd FJ (2004). Accommodative coping and well-being of midlife parents of children with mental health problems or developmental disabilities. Am J Orthopsychiatry..

[10] Singer GH (2006). Meta-analysis of comparative studies of depression in mothers of children with and without developmental disabilities. Am J Ment Retard..

[11] Mori K, Ujiie T, Smith A (2009). Parental stress associated with caring for children with Asperger's syndrome or autism. Pediatr Int..

[12] Emerson E, Llewellyn G (2008). The mental health of Australian mothers and fathers of young children at risk of disability. Aust N Z J Public Health..

[13] Al-Eithan MH, Robert AA, Al-Saeed AH (2010). Mood problems of mothers with disabled children in Saudi Arabia. A preliminary prospective study. Saudi Med J..

[14] Al-Eithan MH, Al Juban HA, Robert AA (2012). Alexithymia among Arab mothers of disabled children and its correlation with mood disorders. Saudi Med J..

[15] Carlson EB, Putnam FW (1993). An update on the Dissociative Experiences Scale. Dissociation..

[16] Zigmond AS, Snaith RP (1983). The hospital anxiety and depression scale. Acta Psychiatr Scand..

[17] Tomkiewicz S (1987). The life of parents with handicapped children. Pediatrie..

[18] Kazak AE (1987). Families with disabled children: stress and social networks in three samples. J Abnorm Child Psychol..

[19] Molsa PK, Ikonen-Molsa SA (1985). The mentally handicapped child and family crisis. J Ment Defic Res..

[20] Blacher J, Nihira K, Meyers CE (1987). Characteristics of home environment of families with mentally retarded children: comparison across levels of retardation. Am J Ment Defic..

[21] Bebko JM, Konstantareas MM, Springer J (1987). Parent and professional evaluations of family stress associated with characteristics of autism. J Autism Dev Disord..

[22] Firat S, Diler RS, Avci A (2002). Comparison of psychopathology in the mothers of autistic and mentally retarded children. J Korean Med Sci..

[23] Hodapp RM, Dykens EM, Masino LL (1997). Families of children with Prader-Willi syndrome: stress-support and relations to child characteristics. J Autism Dev Disord..

[24] Piven J, Chase GA, Landa R (1991). Psychiatric disorders in the parents of autistic individuals. J Am Acad Child Adolesc Psychiatry..

[25] Veisson M (1999). Depression symptoms and emotional states in parents of disabled and non-disabled children. J Soc Behav Pers..

